# Point‐of‐Care Testing by Multiplex‐PCR in Different Compartments in Suspected Lower Respiratory Tract Infection After Lung Transplantation—Results of a Prospective Study

**DOI:** 10.1111/tid.70036

**Published:** 2025-04-26

**Authors:** Susanne Simon, Merle Sophie Kaiser, Marcus Bachmann, Gérard Krause, Jens Gottlieb

**Affiliations:** ^1^ Department of Respiratory Medicine and Infectious Diseases Hannover Medical School Hannover Germany; ^2^ Department of Epidemiology Helmholtz Centre for Infection Research Braunschweig Germany; ^3^ German Centre for Infection Research (DZIF) partner site Braunschweig Germany; ^4^ TWINCORE Centre for Experimental and Clinical Infection Research a joint venture of the Hannover Medical School and the Helmholtz Centre for Infection Research Hannover Germany; ^5^ Biomedical Research in Endstage and Obstructive Lung Disease Hannover (BREATH) Member of the German Center for Lung Research (DZL) Hannover Germany

**Keywords:** lung transplantation, microbiological techniques, molecular diagnostic techniques, multiplex‐polymerase chain reaction, nucleic acid amplification techniques, point‐of‐care systems, respiratory tract infection

## Abstract

**Background:**

Respiratory tract infections (RTIs) are a leading cause of morbidity and mortality following lung transplantation (LTx). This study evaluated a point‐of‐care multiplex‐PCR testing system (POCTmPCR) for pathogen detection in various respiratory samples from LTx recipients.

**Methods:**

In a prospective single‐center study, LTx recipients with RTI undergoing bronchoscopy were enrolled. Samples from bronchoalveolar lavage (BAL), sputum, and nasopharyngeal swabs (NPS) were analyzed by POCTmPCR in conjunction with conventional diagnostics. The primary study endpoint was the concordance of POCTmPCR results between samples (DRKS00032359).

**Results:**

Fifty participants with a median age of 48 years were included; 28 (56%) were previously colonized. Using POCTmPCR, 44 bacterial pathogens were identified in BAL from 30 patients, 49 in sputum (30 patients), and 33 in NPS (17 patients). POCTmPCR identified 24 viral pathogens in BAL from 20 patients, 22 pathogens in sputum of 19 patients, and 19 in NPS of 19 patients. For viral POCTmPCR, sensitivity and specificity compared to BAL were 84% and 97% in sputum, and 80% and 97% in NPS, respectively. For bacterial POCTmPCR, sensitivity and specificity were 80% and 67% in sputum, and 37% and 85% in NPS, respectively. POCTmPCR in comparison to conventional workup had a sensitivity of 89% and 80% and specificity of 75% and 76% for viral and bacterial pathogens, respectively.

**Conclusion:**

POCTmPCR in nasal swabs and sputum may serve as an alternative to BAL for detecting respiratory viruses. Performance for bacterial detection in noninvasive samples was lower. The POCTmPCR system used lacks detection for SARS‐CoV‐2 and *Aspergillus* spp.

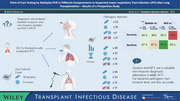

## Graphical abstract

Use of POCtmPCR with noninvasive samples (NPS and sputum) in LTx recipients with viral LRTI are a viable alternative to invasive BAL sampling,

**Abbreviations**
BAL: bronchoalveolar lavage
CAP: community‐acquired pneumonia
CLAD: chronic lung allograft dysfunction
hMPV: human metapneumovirus
LRTI: lower respiratory tract infection
LTx: lung transplantation
MRGN: multi‐resistant Gram‐negative bacteria
NPS: nasopharyngeal swab
NPV: negative predictive value
PCR: polymerase chain reaction
POCTmPCR: point‐of‐care testing by multiplex‐polymerase chain reaction
PPV: positive predictive value
RSV: respiratory syncytial virus
RTI: respiratory tract infection
SARS‐CoV‐2: severe acute respiratory syndrome coronavirus type 2

## 1 Introduction

The first lung transplantation (LTx) was first performed in 1963 and has since been an established treatment for patients with end‐stage lung disease [1]. Although survival rates have improved in recent years, chronic lung allograft dysfunction remains the leading cause of post‐LTx mortality [2], followed by infections, particularly of the lower respiratory tract [3]. Lung transplant recipients are at high risk for opportunistic fungal and bacterial infections, necessitating early diagnosis and preemptive treatment of lower respiratory tract infections (LRTIs) [4, 5]. Bronchoscopy and bronchoalveolar lavage (BAL) are currently regarded as essential components in the diagnostic workup for suspected lower airway infections.

Bronchoscopy with bronchoalveolar lavage is a key diagnostic tool for suspected lower airway infections and is widely used in both inpatient and outpatient settings. However, as an invasive procedure, it carries risks such as bleeding, respiratory failure, and pneumothorax [6]. Additionally, bronchoscopy may not always be feasible due to factors such as patient condition, fasting status, or logistical constraints, making noninvasive sampling necessary [7]. Nasal swabbing and saliva collection are the primary noninvasive alternatives, yet studies evaluating their diagnostic utility in LTx patients remain scarce.

Beyond selecting an appropriate sampling method, rapid and accurate pathogen identification is critical for optimizing treatment. Traditional culture‐based diagnostics are time‐consuming and labor‐intensive, often necessitating empirical treatment before results are available. Multiplex‐PCR‐based point‐of‐care testing (POCTmPCR) offers a promising alternative by enabling early detection of bacterial and viral pathogens in LRTIs. The term point‐of‐care testing (POCT) refers to diagnostic examinations performed in medicine outside a central laboratory, directly at the patient's bedside, or in hospital wards. Studies suggest that POCTmPCR can reduce laboratory turnaround times, facilitating faster and more targeted treatment, particularly when antimicrobial resistance genes are directly detected [8, 9]. However, the clinical benefits of rapid‐response multiplex‐PCR remain a topic of debate [9, 10, 11, 12, 13, 14, 15].

This study aimed to evaluate the efficacy of POCTmPCR in identifying respiratory pathogens across various airway compartments in lung transplant recipients, incorporating noninvasive diagnostic methods such as sputum sampling and nasopharyngeal swabs (NPS).

## 2 Methods

### 2.1 Study Design

We performed a longitudinal, observational, prospective, single‐center cohort study between March 2022 and July 2022. LTx recipients with suspected LRTI or known graft‐colonization visiting a specialized outpatient clinic located at Hannover Medical School were enrolled. The outpatient clinic is a high‐volume LTx center with more than 1000 patients in follow‐up. BAL was subject to conventional diagnostic methods such as quantitative bacterial culture techniques, or multiplex viral PCR, as described below. In conjunction, BAL, sputum, and NPS were analyzed with the Biofire Pneumonia Plus Panel (product code: RFIT‐ASY‐0143, Biomerieux, France). The primary endpoint of this study was the agreement of POCTmPCR results from BAL and two other compartments (NPS, sputum). Secondary endpoint was the comparison of POCTmPCT to conventional methods in BAL.

The Biofire Pneumonia Plus Panel, manufactured by BioMérieux, is a multiplex POCT PCR test. While it detects a total of 18 bacteria (11 Gram‐negative, four Gram‐positive, and three atypical), seven antibiotic resistance markers, and nine viruses that can cause pneumonia and other (lower) respiratory symptoms, its diagnostic spectrum lacks however fungi, especially *Aspergillus* and *Pneumocystis jirovecii*, mycobacteria and severe acute respiratory syndrome coronavirus type 2 (SARS‐CoV‐2) (Table S1). Its major advantage next to the broad spectrum of detectable pathogens and the inclusion of multiple antibiotic resistance genes in the test panel is its short analysis time of 70 min, with only 5–10 min hands‐on time for sample preparation. Sample throughput is limited as the test is carried out using the Biofire Filmarray system [16], which allows only one sample per assay run, but includes all nucleic acid amplification techniques processing steps such as nucleic acid isolation, cDNA synthesis, and PCR amplification in one unit.

The study was performed according to the Declaration of Helsinki from 1975 and according to the standards of the 2008 Declaration of Istanbul. The study was approved by the local ethics committee (10098_BO‐S_2021) and registered at the German Clinical Trials Register (ID DRKS00032359). All patients provided written informed consent, no financial compensation was offered for participating in the study.

### 2.2 Inclusion Criteria

All LTx recipients aged 18 years and above with suspected LRTI or known graft‐colonization as defined below presenting to the outpatient clinic with an existing indication for bronchoscopy with BAL were eligible. A respiratory tract infection was defined as at least two criteria of the following: acute feeling of illness or fatigue, new or increasing opacities on chest x‐ray, new hypoxemia (SpO_2_ or SaO_2 _< 92%, paO_2 _< 60 mmHg, or need for oxygen), body temperature greater than 38°C within the last 7 days, C‐reactive protein (CRP) of ≥30 mg/L, or procalcitonin above 0.5 µg/L. Respiratory tract colonization was defined as at least one positive culture from a lower respiratory tract sample with one of the pathogens included in the BIOFIRE Pneumonia Plus Panel in the last 24 months without any signs of acute infection. Exclusion criteria were lack of consent to participate in the study or patients unable to undergo bronchoscopy with BAL.

### 2.3 Study Procedure

After providing written informed consent, the nasal swab was collected first with FlOQ Swabs (Product code: 501CS01 or 502CS01 based on availability, Copan). After swabbing, the tip was broken into a 1 mL UTM‐medium tube (Product code: 350C, Copan) and stored at −20°C until further processing. Next, bronchoscopy with BAL was performed according to international standard with two aliquots of 50 mL of normal saline [17]. Lavage was performed in the lingula or middle lobe or targeted to opacities on chest x‐ray. BAL fluid was pooled and examined in parallel by POCTmPCR, conventional microbiological culture, and conventional virological testing as outlined below. The POCtmPCR test was performed directly in our clinical unit, whereas conventional microbiological and virological diagnostics were conducted in the respective laboratories. After BAL, sputum was collected and frozen at −20°C until further processing. Patients unable to produce sputum underwent sputum induction [18]. Test results of routine workup from diagnostic laboratories were immediately transferred to the hospital information system after validation and treating physicians were immediately informed about POCTmPCR results. As per our standard clinical procedure, transbronchial biopsy to rule out acute allograft rejection was performed if endobronchial findings were incompatible with infection. All procedures were carried out following the standard operation procedure implemented at Hannover Medical School.

### 2.4 Conventional Diagnostics

As per our standard protocol, BAL samples were assessed by conventional viral duplex PCR for the presence of nucleic acid from adenovirus, human metapneumovirus (hMPV), influenza virus type A and B, parainfluenza virus type 1–4, rhinovirus, human endemic corona viruses (229E, NL63, OC43), and respiratory syncytial virus (RSV A/B) on an ABI 7500 system (Applied Biosystems) with the Respiratory Multi Well System MWS R‐GENE Duplex PCR Kits (BioMérieux). SARS CoV‐2 NAAT was performed with ARGENE.

SARS‐CoV‐2 R‐GENE Kit from BioMérieux to detect the presence of herpes simplex virus (HSV) and rhinovirus (RV) infection was analyzed with the respective R‐Gene Kits from BioMérieux. Whole blood was used in all patients to check for *Cytomegalovirus* infection with the R‐Gene Kit from BioMérieux.

The standard bacterial culture media included Thayer‐Martin agar (Oxoid), MacConkey agar (MAST), Columbia blood agar (BD BBL), chocolate agar (BD BBL), Mannit‐NaCl‐agar (BD), and tryptic soy broth as enrichment medium (Oxoid). For bacterial and yeast identifications, we used Vitek identification cards (Vitek2‐XL, BioMérieux) or MALDI‐TOF (BioMérieux Vitek‐MS with Saramis database). For mycological cultures, we used malt extract agar (Oxoid). Mycobacterial testing was performed using Löwenstein–Jensen–Medium (BioMérieux), Stonebrink Medium (Oxoid), and MGIT liquid medium (BD). Direct microscopy was performed using Gram and Auramin stain for BAL. Molds were identified via sequencing of the internal transcribed spacer region or via microscopy. Antibiotic susceptibility was tested with the Merlin MICRONAUT microdilution system (Merlin Diagnostika) or the Vitek‐2 XL system (BioMérieux).

### 2.5 Statistical Analysis

Sample size calculation was based on the results of the DBATE‐IT study [8], where the sensitivity and specificity of BAL‐PCR were found to be 80%. With a prevalence of 20%, an alpha of 0.05, and a power of 0.8, a minimum number of 49 cases were calculated. If a technical device failure rate of 2% is assumed as drop‐out rate, we aimed for a sample size of 50.

The primary endpoint of the study was the detection rate of bacterial and viral LRTI pathogens by the Pneumonia Plus Panel in NPS and sputum compared to BAL.

Statistical analysis was performed using SPSS v27 (IBM SPSS Statistics,). Categorical variables are presented as numbers (*n*) and percentages (%), continuous variables as median and 25th and 75th quartiles or percentiles. Agreement between POCTmPCR and conventional testing was assessed by calculating sensitivity, specificity, negative predictive value (NPV), positive predictive value (PPV), and diagnostic accuracy. Pathogens detected by conventional tests not included in POCTmPCR were not taken into account in analysis of test performance. Organisms belonging to the normally lower respiratory tract flora or without lung‐pathogenicity (e.g., *Candida* spp., enterococci, coagulase‐negative staphylococci, *Neisseria*, *Penicillium*) were not regarded as positive results.

## 3 Results

### 3.1 Patient Characteristics

Fifty‐one LTx recipients were included between March and July 2022. One patient was excluded from further analysis due to an infection with *Mycobacterium tuberculosis*. Enrolled patients were mainly bilateral lung transplant recipients (96%), two patients received a combined liver–lung transplant. The most common underlying disease was cystic fibrosis. More than half of the patients were colonized, most of them with *Pseudomonas aeruginosa* (32%) or *S. aureus* (18%). Twenty percent of patients were affected by chronic lung allograft dysfunction (CLAD); characteristics are summarized in Table 1.

**TABLE 1:** Patient characteristics.

	*N* = 50
Gender female, *n* (%)	21 (42)
Age (years), median (25%, 75% percentile)	48 (36, 59)
CRP (mg/L), median (25%, 75% percentile)	1.9 (0.8, 26.5)
Body mass index (kg/m^2^), median (25%, 75% percentile)	22.0 (18.9, 24.2)
Glomerular filtration rate (mL/min/1.73 m^2^), median (25%, 75% percentile)	44 (39, 70)
Chronic lung allograft dysfunction, *n* (%)	10 (20)
Transplant type, *n* (%)	
Bilateral	48 (96)
Combined liver lung transplantation	2 (4)
Underlying disease, *n* (%)	
Cystic fibrosis/bronchiectasis	21 (42)
Emphysema incl. alpha1ATD	11 (22)
Fibrosis/interstitial lung disease	14 (28)
Other	4 (8)
Years after transplantation, median (25%, 75% percentile)	2.0 (0.5, 6.8)
Previous airway colonization, *n* (%)	28 (56)
*Pseudomonas aeruginosa*	16 (32)
*Staphylococcus aureus*	9 (18)
Other	3 (6)
Outpatient bronchoscopy, *n* (%)	48 (96)
Time to pneumonia panel PCR results (minutes), median (25%, 75% percentile)	86 (77, 110)
Transbronchial biopsy performed, *n* (%)	17 (34)
Minimal acute rejection (A1)	4 (8)
Acute rejection (A2+)	2 (4)
Indication for bronchoscopy, *n* (%)	
Airway complication	5 (10)
Infection	17 (34)
Surveillance	20 (40)
Deterioration of graft function	8 (16)

Abbreviations: A1, A2, International Society of Heart and Lung transplantation grading 2007; Alpha1ATD, alpha‐1 antitrypsin deficiency; CRP, C‐reactive protein.

### 3.2 Spectrum of Pathogens Identified in Conventional Workup and POCTmPCR in BAL

Despite a suspected LRTI, 13 (26%) patients had no identifiable pathogen in the POCTmPCR and 15 (30%) patients had none in the conventional diagnostic workup from BAL. The distribution of the pathogen spectrum is shown in Table 2. In conventional BAL, 42 bacterial pathogens were identified in 30 patients, most frequently *Pseudomonas* spp. (*n* = 16, 32%), followed by *S. aureus* (*n* = 10, 20%). In seven patients, *Aspergillus* was detectable in conventional cultures from BAL. A direct comparison was limited as the POCTmPCR does not contain primers to detect nucleic acid from *Aspergillus* species. Similarly, a direct comparison was not possible for SARS‐CoV‐2. Four SARS‐CoV‐2 infections were identified by conventional duplex PCR. In BAL, a total of 24 respiratory viruses were identified in POCTmPCR of 20 patients, and 16 viruses were identified by conventional PCR tests in 14 patients. Samples from 21 patients were only subject to conventional triplex PCR for SARS‐CoV‐2, influenza A/B, and RSVA/B, limiting also the comparability. Interestingly, no RSV infection was detected in either conventional or POCT testing from BAL.

**TABLE 2:** Identified pathogens in the different respiratory departments by conventional, POCTmPCR‐BAL, ‐sputum, and ‐swab.

Species	Conventional BAL *n*	POCTmPCR‐BAL *n*	POCTmPCR‐sputum *n*	POCTmPCR‐NPS *n*
**Bacteria**				
*Pseudomonas* spp.	16	18	15	9
*Staphylococcus aureus*	10	12	18	5
*Klebsiella pneumoniae*	2	2	2	1
*Aspergillus* spp.	7	n.a.	n.a.	n.a.
*Escheria coli*	3	3	2	—
*Haemophilus influenzae*	3	2	3	3
*Moraxella catarrhalis*	1	4	4	3
*Streptococcus agalactiae*	—	1	1	1
*Serratia marcesens*	—	—	1	—
*Enterobacter cloacae*	—	1	1	—
*Proteus mirabilis*	—	—	1	—
*Streptococcus pneumoniae*	—	1	1	1
No bacterial pathogen	17	20	20	33
Bacterial co‐infection	9	9	15	4
Total bacterial pathogens	42	44	49	23
**Viruses**				
Influenza	2	2	2	2
Respiratory syncytial virus	—	—	—	—
Human pneumovirus	3	4	4	3
Coronavirus (excl. SARS‐CoV‐2)	2	3	2	3
Parainfluenza	2	3	3	2
Rhino‐/Enterovirus	3	11	19	9
MERS‐CoV	—	—	—	—
Adenovirus	—	1	1	—
SARS‐CoV‐2	4	n.a.	n.a.	n.a.
No viral pathogen	36	30	31	31
Viral co‐infection	3	4	3	0
Total viral pathogens	16	24	22	19
Bacterial and viral co‐infection	14	13	12	6
No respiratory pathogen at all	15	13	14	21

Abbreviations: BAL, bronchoalveolar lavage; MERS‐CoV, Middle East respiratory syndrome coronavirus; NPS, nasopharyngeal swab; n.a., not applicable; POCTmPCR, point‐of‐care testing multiplex‐polymerase chain reaction; SARS‐CoV‐2, severe acute respiratory syndrome coronavirus type 2.

**FIGURE 1:**
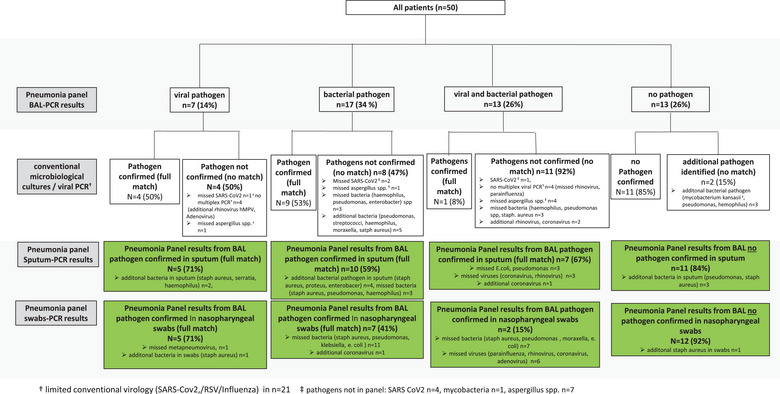
Agreement of bacterial and/or viral pathogen for BAL POCTmPCR in comparison to sputum, NPS POCTmPCR, and conventional tests. BAL = bronchoalveolar lavage, NPS = nasopharyngeal swab, PCR = polymerase chain reaction, POCTmPCR = point‐of‐care testing multiplex‐polymerase chain reaction, SARS‐CoV‐2 = severe acute respiratory syndrome coronavirus type 2.

The agreement of bacterial and/or viral pathogen for BAL POCTmPCR in comparison to sputum, NPS POCTmPCR, and conventional tests is shown in Figure 1.

### 3.3 Sensitivity and Specificity POCTmPCR BAL Versus POCTmPCR Sputum and Swabs

Table 3 shows the results of the diagnosis of POCTmPCR from BAL compared with the diagnosis of POCTmPCR from sputum and NPS. Diagnostic performance of sputum and NPS in comparison to BAL was acceptable for viral pathogens but limited for bacterial pathogens.

**TABLE 3:** Diagnostic test evaluation of pneumonia panel PCR results in sputum and swabs.

	POCTmPCR in sputum samples		POCTmPCR in nasopharyngeal swabs	
	vs. POCTmPCR viral panel in BAL	vs. POCTmPCR bacterial panel in BAL	vs. POCTmPCR viral panel in BAL	vs. POCTmPCR bacterial panel in BAL
No matching results	2%	16%	6%	36%
Partial matching results	6%	12%	6%	6%
Full matching results	92%	72%	88%	58%
Sensitivity	84%	80%	80%	37%
Specificity	97%	67%	97%	85%
Positive predictive value	94%	72%	94%	79%
Negative predictive value	91%	76%	88%	47%
Accuracy	92%	74%	90%	56%

Abbreviations: BAL, bronchoalveolar lavage; PCR, polymerase chain reaction; POCTmPCR, point‐of‐care testing multiplex‐polymerase chain reaction.

### 3.4 Sensitivity and Specificity of POCTmPCR Versus Conventional Diagnostics

Table S2 demonstrates diagnostic test evaluation of POCTmPCR versus conventional BAL diagnostics. POCTmPCR showed slightly better sensitivity and NPV compared to conventional virological diagnostics compared to bacterial diagnostics (89% vs. 80% and 90% vs. 79%), and specificity, PPV, and accuracy were similar (see Table S2).

### 3.5 Antibiotic Resistance Markers

In BAL, POCTmPCR identified two *S. aureus* pathogens with resistance gene mecA/C and MREJ, four were identified in sputum POCTmPCR (two were concordant), no resistance genes were identified in swabs. A single POCTmPCR‐positive resistance gene was identified as methicillin‐resistant *S. aureus* in cultures of BAL fluid.

## 4 Discussion

To our knowledge, this is the first study to compare the performance of POCTmPCR across different respiratory compartments in immunocompromised patients with RTI. In our analysis, POCTmPCR applied to NPS and sputum demonstrated high accuracy (90% and 92%, respectively) and a specificity of 97% for respiratory viruses compared to invasive BAL. However, its utility for detecting bacterial pathogens in noninvasive samples was limited.

We found no previous studies directly comparing POCTmPCR performance between BAL and noninvasive samples. However, some studies have evaluated conventional diagnostics against POCTmPCR, as outlined in our secondary endpoint. The Unyvero Pneumonia Panel detects 20 bacterial species, *P. jirovecii*, and 16 resistance genes, while the FilmArray panel identifies 18 bacteria, eight resistance genes, and nine viral pathogens. A recent study assessed this POCTmPCR system against conventional diagnostics in 6523 specimens from the lower respiratory tract in 15 hospitals [14]. PCR methods detected significantly more pathogens than culture (Unyvero: 60.4%, FilmArray: 74.2% vs. culture: 44.2%), a finding corroborated by our study, particularly in BAL, where POCTmPCR identified more viruses (24 vs. 16).

Additionally, a prospective study of 605 patients, over half of whom were immunocompromised, including 16% of lung transplant recipients, further supports these findings [12]. This study used a different panel (Curetis Unyvero ‐ P50 assay) than ours. While multiplex‐PCR detected pathogens more frequently than culture (82% vs. 56%), its use did not impact hospitalization duration or antibiotic therapy. Immunocompetent patients had higher detection rates in both culture and PCR compared to immunosuppressed patients, likely because the panel does not cover rare pathogens. The reported sensitivity for bacterial multiplex‐PCR was 81%, with a specificity of 87%, which was higher than the specificity observed in our study (76%).

NPS proved useful for noninvasive RTI diagnostics in our study, with a PPV of 94% and an NPV of 88%. A separate study on SARS‐CoV‐2 testing in BAL versus NPS in 128 critically ill patients with respiratory failure reported similar findings, demonstrating high concordance [19]. Additionally, other research confirmed that NPS has a strong PPV (86%) and NPV (94%) for viral diagnostics compared to BAL [20]. However, these studies did not utilize multiplex‐PCR, though their findings support the value of noninvasive NPS as an alternative to invasive BAL sampling.

A multiplex‐PCR analysis of NPS from 200 hospitalized COVID‐19 patients screened for bacterial co‐infections and non‐SARS‐CoV‐2 viruses [21]. While 43% tested positive, there was no significant impact on clinical outcomes such as mortality or hospital stay, highlighting the limited sensitivity of bacterial diagnostics from NPS in our study (37%).

As a noninvasive sample, sputum was used by Gadsby et al. with a rapid multiplex real‐time PCR to diagnose community‐acquired pneumonia (CAP) in hospitalized patients. PCR detected bacteria in 78% of cases, significantly more than in culture (32%, *p* < 0.001) [22]. Similarly, our study showed that POCTmPCR identified more bacterial pathogens in sputum than conventional BAL analysis (49 vs. 42 total bacterial pathogens).

In a study of 142 children, paired sputum and BAL samples analyzed via multiplex‐PCR had similar positivity rates (BAL: 86% vs. sputum: 80%), with higher consistency for viral than bacterial pathogens—findings consistent with our study [23]. Additionally, a Chinese study using multiplex‐PCR on sputum and oropharyngeal swabs identified more viruses than bacteria overall, with sputum yielding better bacterial detection than swabs [24]. This may be due to contamination from oropharyngeal flora, which we accounted for by excluding nonpathogenic microorganisms.

National German guidelines do not recommend molecular diagnostics for community‐ or hospital‐acquired pneumonia [10, 11] when a bacterial pathogen is likely, aligning with our finding that NPS had the lowest sensitivity (37%) and accuracy (56%) for bacterial detection.

Multiplex‐PCR for resistance gene detection has an estimated error rate of 30%, particularly with *P. aeruginosa* [14]. In our study, POCTmPCR proved unreliable for detecting resistance genes. Although 32% (*n* = 16) of patients were colonized with *P. aeruginosa*, including one with 4MRGN (multi‐resistant Gram‐negative bacteria) and three with 3MRGN, no carbapenemase‐encoding resistance genes were identified by multiplex‐PCR.

Multiplex‐PCR is the gold standard for detecting community‐acquired respiratory viruses. A large meta‐analysis of 27 studies involving 17,321 patients found that PCR results were available 24 h earlier than conventional methods (viral culture, immunofluorescence assays, single‐target RT‐PCR) and reduced hospital stay by nearly 1 day [9].

The rapid turnaround time and high sensitivity of viral multiplex‐PCRs are particularly beneficial for lung transplant patients, as early detection can guide timely antiviral treatment (e.g., oseltamivir, remdesivir). Clark et al. demonstrated that patients diagnosed with influenza via PCR were more likely to receive antiviral therapy (RR 1.55, 95% confidence interval: 1.16–2.07) [9]. Similarly, the DBATE study found that multiplex‐PCR led to a relevant therapeutic intervention in 10 out of 60 lung transplant patients and reduced the turnaround time by 1 day [8].

In our study, 10 out of 50 patients received antiviral therapy. POCTmPCR would significantly reduce the time to treatment compared to conventional diagnostics, further supporting its clinical utility.

Few prospective studies have assessed the impact of molecular diagnostics on patient‐relevant outcomes. While these techniques can increase pathogen detection rates to 90%, they have not demonstrated clear improvements in clinical outcomes [25, 26]. Multiple pathogens are frequently identified; in our study, more than 20% of patients had two or more pathogens detected [26].

Bronchoscopy is a key diagnostic tool for suspected LRTIs in lung transplant patients and plays a crucial role in detecting acute rejection, fungal infections, and airway complications such as insufficiencies or central airway obstruction [27, 28]. Direct visualization and additional sampling, including transbronchial biopsies, provide significant diagnostic value.

In hematologic patients, a randomized controlled trial demonstrated that noninvasive diagnostics were non‐inferior to bronchoscopy [7]. Similarly, a retrospective study of 200 ICU patients with hospital‐acquired pneumonia compared invasive (BAL or bronchoscopic aspirate) and noninvasive (sputum or endotracheal aspirate) diagnostics, finding that invasive methods led to higher pathogen detection (56% vs. 39%, *p* = 0.018) and more targeted therapy [29]. While noninvasive approaches offer advantages in convenience and accessibility, invasive specimen collection remains essential in certain cases.

Our study has several limitations. The Biofire Pneumonia Plus Panel has a restricted pathogen spectrum for lung transplant patients. Conventional diagnostics detected *Aspergillus* in seven cases and SARS‐CoV‐2 in three cases, neither of which are included in the panel. Given the high risk of fungal infections in LTx patients, targeted diagnostic approaches are necessary. Future specialized panels for immunocompromised patients and specific clinical indications, such as CAP, could enhance diagnostic accuracy and patient management [21, 25, 26].

## 5 Conclusion

POCTmPCR in NPS and sputum appears to be a valuable noninvasive tool for detecting respiratory viruses in LTx recipients, with performance comparable to conventional viral PCR. However, its reliability in identifying bacterial pathogens is limited, particularly in noninvasive samples. Given the high prevalence of bacterial infections in lung transplant patients, bronchoscopy and conventional cultures remain the gold standard. POCT multiplex‐PCR systems offer shorter turnaround times, but randomized controlled trials are needed to assess their impact on patient outcomes more comprehensively.

## Author Contributions

Conception and design: JG, SS. Administrative support: SS, JG. Provision of study materials or patients: all authors. Collection and assembly of data: SS, MK, JG, MB. Data analysis and interpretation: JG, SS. Manuscript writing: JG, SS. Final approval of manuscript: all authors.

## Conflicts of Interest

Susanne Simon reports speaker fees from Gilead and consultancy fees from Pfizer and advanz. All disclosures are unrelated to the current work. Jens Gottlieb reports institutional research grants from Zambon/Breath Therapeutics, German Center of Lung Research, Deutsche Forschungsgemeinschaft. He also received fees for advisory/consultancy from Theravance, Pierre Fabre, Atheneum, Merck, Springer Healthcare, European Research Network, and speaker fees from Novartis, Astra Zeneca, CSL Behring. He owns stock options of Pfizer. He serves as a member of the ScanCLAD study´s data safety monitoring board. All disclosures are unrelated to the current work. Merle Sophie Kaiser, Marcus Bachmann, and Gerard Krause declare no conflicts of interest.

## Supporting information



Supporting Information

Visual Abstract

## Data Availability

Research data are not shared.
