# Supplementary material for: Point‐of‐Care Testing by Multiplex‐PCR in Different Compartments in Suspected Lower Respiratory Tract Infection After Lung Transplantation—Results of a Prospective Study
Source: Transpl Infect Dis. 2025 Apr 26;28(1):e70036. doi: 10.1111/tid.70036 (PMC12892833; doi:10.1111/tid.70036)
Supplement: Supplementary file 1 — Supporting Information [file TID-28-e70036-s001.docx]

Supplementary Material

**Table S1: Diagnostic Spectrum of the Pneumonia Panel plus (Biomerieux, Marcy-Étoile, France)**

| **Bacteria** | **Antibiotic resistance genes** |
| --- | --- |
| Acinetobacter calcoaceticus-baumannii complex  Enterobacter cloacae  Escherichia coli  Haemophilus influenzae  Klebsiella aerogenes  Klebsiella oxytoca  Klebsiella pneumoniae group  Moraxella catarrhalis  Proteus spp.  Pseudomonas aeruginosa  Serratia marcescens  Staphylococcus aureus  Streptococcus agalactiae  Streptococcus pneumoniae  Streptococcus pyogenes  Legionella pneumophila  Mycoplasma pneumoniae  Chlamydia pneumoniae | ESBL  CTX-M  Carbapenemases  KPC  NDM  Oxa48  VIM  IMP  Methicillin Resistance mecA/mecC and MREJ |
| **Viruses** | |
| Influenza A Influenza B  Adenovirus  Coronavirus (non SARS-CoV2)  Parainfluenzavirus  Respiratory Syncytialvirus  Human Rhinovirus/Enterovirus  Human Metapneumovirus  Middle East Respiratory Syndrome Coronavirus (MERS-CoV) | |

KPC - Klebsiella pneumoniae carbapenemase, NDM - New Delhi metallo-β-lactamase, Oxa48 – Oxacillinase-48 like carbapenemase, VIM - Verona integron-encoded metallo-β -lactamase, IMP - Imipenemase metallo-beta-lactamase, mecA/mecC – resistance genes encoding the penicillin-binding protein 2A, MREJ – mec right extremity junction, CTX-M – Cefotaximase-Munich, EBSL – extended spectrum betalaktamases, SARS-CoV2 - Severe acute respiratory syndrome coronavirus type 2

**Table S2: Diagnostic test evaluation of Pneumonia Panel PCR Results after exclusion of pathogens not included in panel (Aspergillus, SARS-COV 2)**

|  | POCTmPCR in BAL | |
| --- | --- | --- |
|  | Vs. conventional virology* | Vs. conventional microbiology |
| No matching results | 10% | 10% |
| Partial matching results | 14% | 14% |
| Full matching results | 76% | 76% |
| Sensitivity | 89% | 80% |
| Specificity | 75% | 76% |
| Positive predictive value | 72% | 77% |
| Negative predictive value | 90% | 79% |
| Accuracy | 81% | 80% |

BAL – bronchoalveolar lavage, PCR – polymerase chain reaction, POCTmPCR = point of care testing multiplex-polymerase chain reaction, SARS-CoV2 severe acute respiratory syndrome coronavirus type 2, * 21 patients were only subject to triplex PCR for SARS-CoV-2, Influenza A/B and RSVA/B
