# Supplementary material for: Point‐of‐Care Testing by Multiplex‐PCR in Different Compartments in Suspected Lower Respiratory Tract Infection After Lung Transplantation—Results of a Prospective Study
Source: Transpl Infect Dis. 2025 Apr 26;28(1):e70036. doi: 10.1111/tid.70036 (PMC12892833; doi:10.1111/tid.70036)
Supplement: Supplementary file 2 — Visual Abstract [file TID-28-e70036-s002.pdf]

Point of Care Testing by Multiplex-PCR in Different Compartments in Suspected Lower respiratory Tract Infection LRTI) after Lung Transplantation – Results of a Prospective Study

@TheTxIDJournal

Simon *et al*. ***Transplant Infectious Diseases***. 2025.

Diagnostic concordance between invasive- and non invasive samples via POCTmPCR

50 LTx-Recipients with suspected LRTI

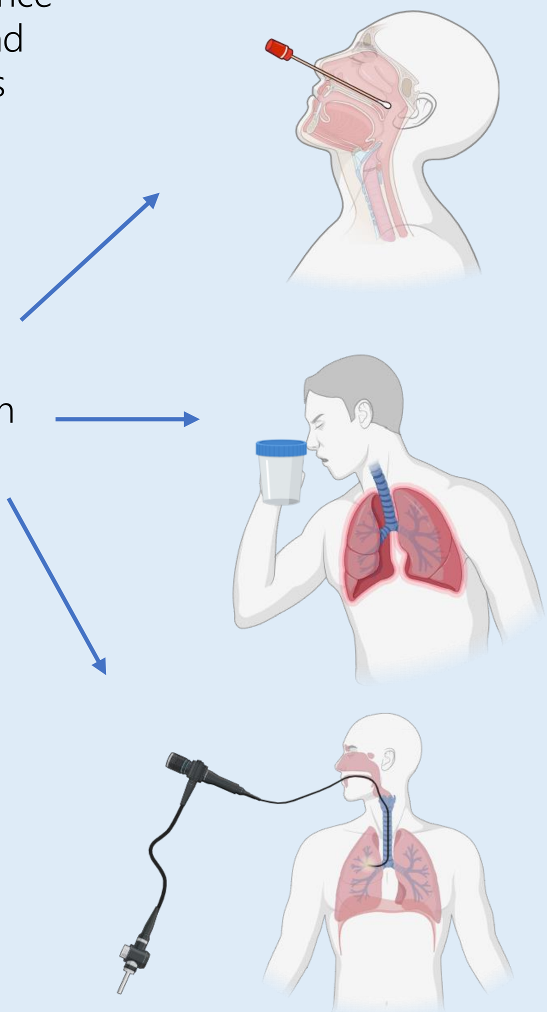

POCTmPCR-Panel

18 bacteria

9 viruses

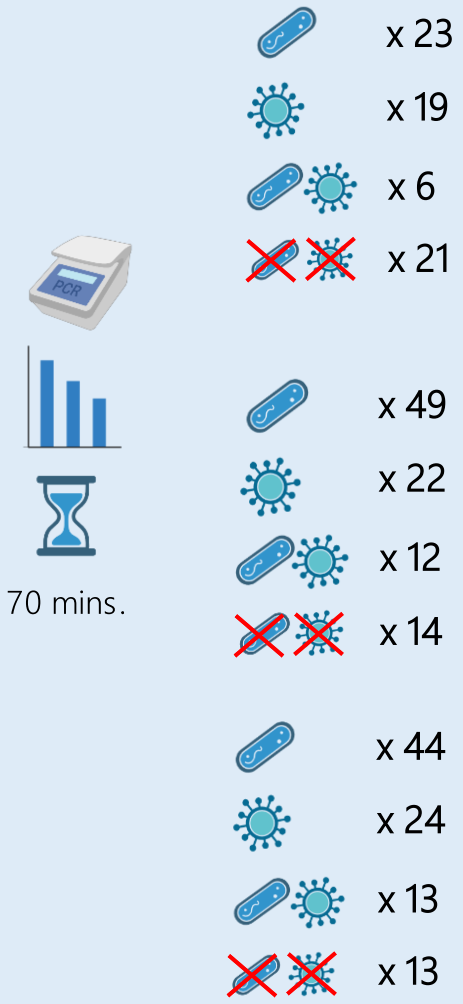

	Sputum vs. BAL (viral)	Sputum vs. BAL (bacterial)	NPS vs. BAL (viral)	NPS vs. BAL (bacterial)
**Sensitivity**	84 %	80%	80 %	37%
**Specificity**	87 %	67 %	97 %	85 %

!

Sputum and NPS are a valuable non-invasive diagnostic alternative in **viral** LRTI.
For bacterial pathogens non-invasive tests are less accurate.
